# Recruiting Chinese Americans With Chronic Conditions Through Electronic Health Records and Community Engagement

**DOI:** 10.1093/geroni/igaf122.1737

**Published:** 2025-12-31

**Authors:** Yaguang Zheng, Susan Zweig, Katharine Lawrence, Keer Chen, Bei Wu

**Affiliations:** New York University, New York, New York, United States; NYU Grossman School of Medicine, New York, New York, United States; NYU Grossman School of Medicine, New York, New York, United States; University of California Health, San Diego, California, United States; NYU Shanghai, Shanghai, China (People’s Republic)

## Abstract

Clinical trials are essential for evaluating the effects of new technologies and treatments on health outcomes; however, varied recruitment approaches may introduce biases that affect the representativeness of study samples, particularly recruiting disproportionately low participation of Asian Americans, including Chinese Americans. This study thus aimed to compare the characteristics of Chinese Americans with chronic conditions recruited through different approaches. In Study I, we recruited 11 Chinese Americans with type 2 diabetes (T2D) via electronic health records, while in Study II, we recruited 11 older Chinese Americans with comorbid T2D and mild cognitive impairment (MCI) through community engagement (e.g., Adult Day Care Center). In Study I, participants’ mean age was 55.6 ± 9.5 years (ranging from 42-77), with 72.7% being male, 81.8% employed, 81.8% at least a college education, 100.0% of family income>$50,000, and 81.8% of primary language being English. In contrast, in Study II, participants had a mean age of 74.5 ± 5.2 years (ranging from 67-86), with 63.6% being female, 100% retired, 36.4% at least a college education, 90.9% family income<$30,000, and 100.0% of primary language being Chinese. Our findings indicate that participants recruited via electronic health records tend to have higher socioeconomic status and fewer language barriers than those recruited through community engagement. Complementary approaches are crucial to recruiting a minoritized population in clinical studies to achieve representativeness. Additionally, linguistically sensitive programs, providers, and healthcare systems are essential for community engagement.

